# Variation of parental feeding practices during the COVID-2019 pandemic: a systematic review

**DOI:** 10.1186/s12889-022-14027-6

**Published:** 2022-08-23

**Authors:** Wen Luo, Qian Cai, You Zhou, Yepeng Cai, Huizi Song, Yiran Zhang, Yuying Chen, Yuexia Liao

**Affiliations:** 1 School of Nursing, School of Public Health, Yangzhou University, Yangzhou, Jiangsu China; 2 Nursing Department, Affiliated Hospital of Yangzhou University, Yangzhou University, Yangzhou, Jiangsu China

**Keywords:** Feeding practices, Feeding behavior, COVID-19

## Abstract

Coronavirus disease 2019 (COVID-19) is an infectious disease caused by severe acute respiratory syndrome coronavirus 2 (SARS-CoV-2), which is highly contagious and has resulted in a protracted pandemic. Infections caused by new coronavirus strains, primarily Delta and Omicron and currently highly prevalent globally. In response to the epidemic, countries, and cities implemented isolation and quarantine guidance, such as limiting social contact, which have affected the lifestyles and quality of life of the population. Parental feeding behaviors may vary as a result of factors such as prolonged home isolation of parents and children, lack of supplies during isolation, and stress. This study was designed to assess the available evidence and its implications for parental feeding practices in the context of COVID-19. We screened and reviewed research published in five electronic databases between 2020 and 2022, and eight studies met the selection criteria. Parents were observed to use a variety of feeding practices, including high levels of coercive control and reduction of rules and limits according to Vaughn’s food parenting constructs. The findings suggest that parental feeding practices are changing as a result of the pandemic and that more research is needed to further explore how to provide supportive feeding guidance to parents during emergencies in order to jointly promote child health.

**Supplementary Information:**

The online version contains supplementary material available at 10.1186/s12889-022-14027-6.

## Introduction

Coronavirus disease (COVID-19), caused by the SARS-CoV-2 virus, was first discovered in Wuhan, China in 2019, causing fever and cough [1]. It is highly contagious and affects a large portion of the world's population. Over the last two years, Delta and Omicron coronavirus variants have been identified, and now there have been over 530,266,292 confirmed cases and 6,299,364 deaths worldwide [2]. In order to control the spread of the virus various governments implemented a range of strategies including home isolation requiring people living through the COVID-19 pandemic to adapt their way of life.

Diet is an important part of a healthy lifestyle for children, and under COVID-19 home isolation, parents are responsible for the majority of their children’s feeding. Parental feeding behaviors are usually assessed in terms of feeding style and feeding practices. Feeding style is a relatively fixed form of behavior that combines parental perceptions, attitudes, behaviors, and emotional expressions concerning feeding their children. Feeding practices are goal-oriented, specific behaviors that parents adopt to influence their children's eating behaviors or intake [3], such as providing a healthy home food environment and modeling eating behaviors that children learn to imitate [4]. For this reason, feeding practices are more susceptible to change during the COVID-19 pandemic. This study categorized parental feeding practices into three categories based on the food parenting practices framework proposed by Vaughn et al. [5]: coercive control, structure, and autonomy support. Coercive control refers to parents imposing their ideas on their children (e.g., children eating when they are not hungry) and controlling them through pressure eating and restriction, and is associated with emotional eating and unhealthy dietary intake (high-fat and high-sugar foods) in children [4, 6, 7], which leads to obesity and disordered eating behaviors in children [8, 9]. Structure refers to the strategies parents used to help influence children's eating behaviors and organize the home environment, representing a type of parental control involving noncoercive practices [5]. The goal of autonomy support is to help children to develop their autonomy and independence in making nutritious choices. Children may benefit from structured practices (e.g., healthy food environments) and autonomy-supportive practices (e.g., praise) that promote healthy dietary intake (e.g., fruits, vegetables) and eating behaviors [10, 11].

Parent–child engagement time has risen as a result of pandemic home isolation measures, as have interactions between children and their parents over food and feeding practices [12]. However, according to the American Psychological Association survey, parents are under tremendous stress [13], possibly as a result of parents working from home, home-schooling, unemployment due to the economic downturn, or food insecurity due to the epidemic. Previous studies have found that different types of stress, such as maternal psychological stress [14], parental emotions [15], food insecurity [16], and parenting stress [17], can affect parental feeding practices. Stressed parents are more likely to exert feeding pressure on their children [18], in particular, parents who experienced stress in the daytime [19], which will influence the child's satiety response [20]. A qualitative study revealed that, despite having different goals for feeding their children (e.g., providing a healthy home food environment, limiting snack intake, etc.), parents are influenced by direct factors (e.g., stress) that make it difficult for them to implement feeding practices as expected [21], which may be a reason for the shift from structured and autonomy-supportive feeding practices to more coercive feeding practices.

It is unknown whether the COVID-19 outbreak or the isolation measures implemented to combat the disease impacts parental feeding practices. Therefore, the purpose of this study was to analyze changes in parental feeding practices during COVID-19 that may help shape future interventions and make parental guidance more targeted.

## Materials and methods

### Search strategy

The Preferred Reporting Items for Systematic Review and Meta-analysis guidelines [22] were followed for this systematic review. Researchers examined the PubMed, EMBASE, CINAHL, MEDLINE, and Web of Science databases for articles published in English between January 2020 and December 2021. To improve the degree of citation retrieval as much as possible, the following MESH subject headings were used as possible: child, child*, adolescent*, teen*, pediatric*, preschool*, feeding behavior, feeding-related behavior*, feeding practice, feeding pattern*, feeding style, etc. and COVID-19, SARS-CoV, coronavirus disease 2019, coronavirus, etc. to describe the epidemic situation. Additional file 1 contains the specific search strategies.

### Inclusion and exclusion criteria

Selected studies included 1) parents of children aged 3–18 and 2) outcome indicators that met the parental feeding practices standards (filtered using Vaughn's framework). Meta-analyses, systematic reviews, reviews, case reports, and qualitative research were excluded since they were irrelevant to the research topic in a nonepidemic context.

### Article screening and data extraction

Two reviewers (Luo and Cai) first performed a brief reading of the title and abstract. Studies that met the inclusion criteria were read in full and assessed for quality criteria. YZ, PYC, and ZHS extracted the following information, which was double-checked by RYZ and QC: 1) Study (authors, year, country), 2) Study population and setting, 3) Tool of assessment, 4) Study variables, 5) Primary Outcome, and 6) Total NOS. Any conflicts were resolved by consensus in a panel discussion dominated by another reviewer (Liao).

### Quality assessment

To better evaluate the literature, the Ottawa–Newcastle (NOS) scale adapted from Herzog [23] was used to evaluate the quality of observational studies. The NOS is composed of three criteria: selection, comparability, and results. The NOS score [24] divides into three levels of quality: low, medium, and high, which are < 5 points, 5– < 8 points, and 8–9 points, respectively. The supplementary material contains the details of the quality assessment.

## Results

### Study characteristics

**Fig. 1:**
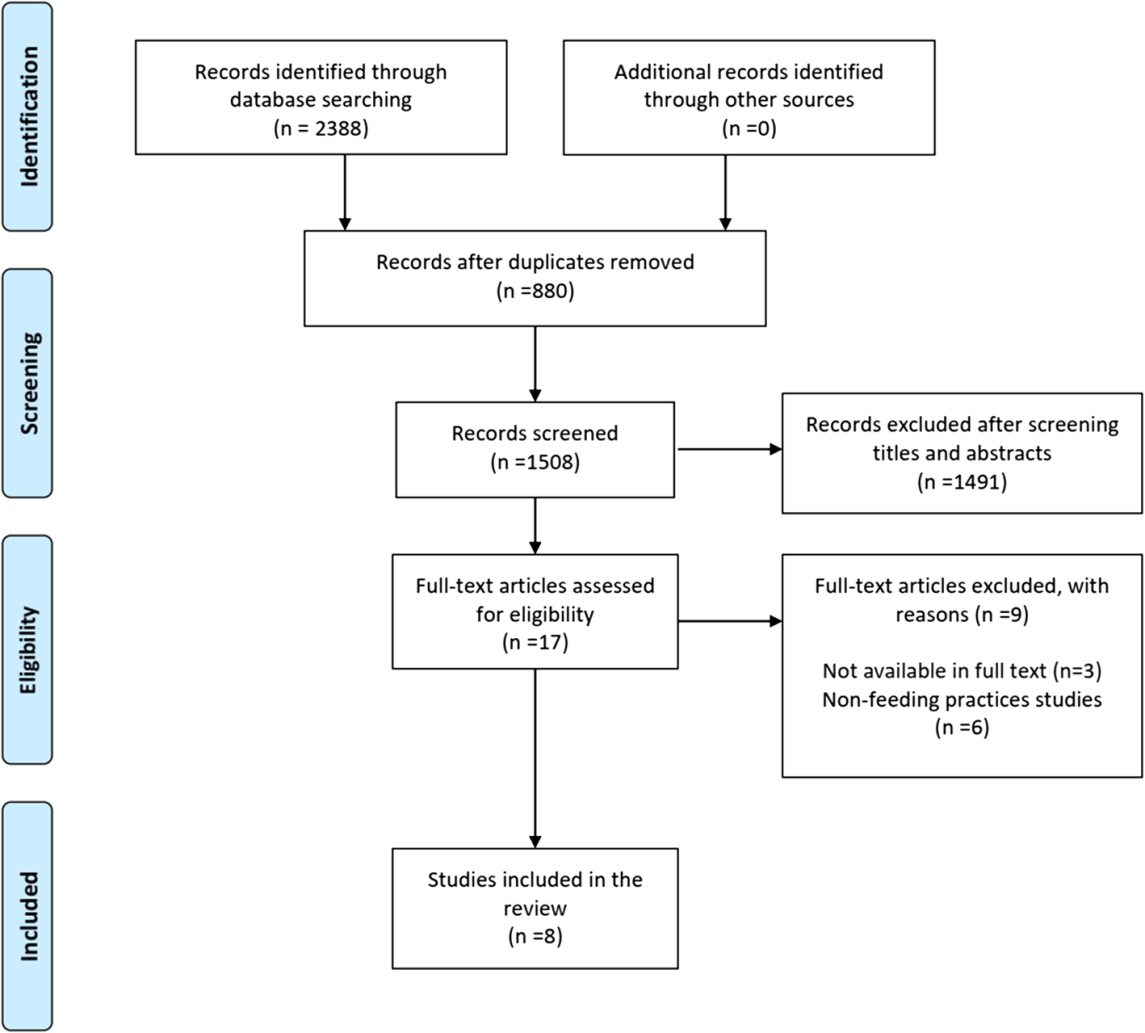
Flow diagram depicting the study search and selection protocol

Figure 1 depicts the article selection flow diagram. Overall, 2388 publications were searched and identified in the database, with 880 being duplicates. After excluding the duplicates based on abstracts and titles, 17 articles were selected for full-text evaluation. Finally, eight publications were considered in this review, six of which were cross-sectional studies and two of which were cohort studies that employed self-reporting measures. The articles by Caroline et al. [25] and Jansende et al. [21]. were classified as high quality by the NOS standard, whereas the remaining six articles [26–31] were classified as medium quality (in Additional File 1). Table 1 shows the study’s characteristics and significant findings.

**Table 1:** Research characteristics and main results

Study (authors, year, country)	Study population and setting	Tool of assessment	Study variables	Primary Outcome	Total NOS
Adams et al., 2020, USA [27]	Select parents of children aged 5–18 years from social media, email using a snowball technique(*n* = 584) From the 30th of April until the 23rd of May 2020	CFQ	Food insecurity, The home food Environment, Parent feeding practices	During COVID-19, Parent-reported use of restrictive feeding practices, pressure to eat, and monitoring also increased restriction, pressure, monitoring; Parents’ increased use of pressure feeding practices was greater for families with low and very low food security, compared with food-secure families; About one-third of families reported an increase in the amount of high-calorie snack foods and desserts/sweets in their home	6
Caroline et al., 2020, USA [25]	Select parents of children aged 7–17 years through the Qualtrics platform(*n* = 189) From June 2020 and February 2021	CFQ	Pandemic-related parental distress, Family meals, Child feeding practices	Higher levels of both parental COVID-19-related impact and distress were associated with lower levels of structured household meals; After controlling for child age and zBMI, both the perceived negative impact of COVID-19 and parent distress related to COVID-19 were positively associated with restriction of child food intake, not associated with pressuring their child to eat more food	9
Adams et al., 2021, USA [28]	Select parents of children aged 5–18 years from social media, email using a snowball technique(*n* = 433) Completed in May 2020 and September 2020	CFQ	Food insecurity, The home food Environment, Parent feeding practices	Parents' use of restriction and pressure to eat increased from before COVID‐19 to T1 (May 2020) and returned to comparable pre‐COVID‐19 values by T2(September 2020). Patterns in parents' reported use of monitoring did not differ by changes in food security status categories	6
Frankel et al., 2021, USA [29]	Select parents of children aged 2–7 years via Facebook posts and targeted advertisements on Facebook and Instagram(*n* = 119) From the periods of mid-April to early June of 2020	FPSQ	COVID‑related parenting stress, Nonresponsive feeding behaviors, Parent mental health	Children’s self-regulation of eating, and COVID-related parenting stress and parent distrust in appetite were both found to decrease children’s ability to self-regulate energy intake	7
Jansen et al., 2021, USA [21]	Select parents of children aged 2–12 years via Amazon’s Mechanical Turk (MTurk) and social media. (*n* = 318) From the 26th of May until the 29th of June 2020	CFPQ FPSQ	Parental stress, Food parenting practices, Child snack intake	Higher COVID-19-specific stress was associated with more non-nutritive use of food and snacks (e. g. emotional and instrumental feeding), but also more structure and positive interactions (e. g. eating with or engaging with child around mealtimes); Higher COVID-19-specific stress was also associated with greater child intake frequency of sweet and savory snacks	9
Loth et al., 2021, USA [26]	Select parents of children aged 2–5 years from Kids EAT! (*n* = 72) Participants completed an online survey, followed by a 10-day EMA at both time points: pre-COVID (October 2019–January 2020) and during COVID (March–April 2020)	EMA	Food-related parenting practices	Most parents of preschoolers use a variety of food parenting practices, including coercive control, indulgence, structure, and autonomy support practice; The use of structured and autonomy supportive practices, however, decreased during the COVID-19 pandemic	6
Philippe et al., 2021, France [31]	Select parents of children aged 3–12 years via an agency disposing of a panel of participants all over France(*n* = 498) From the 30th of April until the 10th of May 2020 (the end of the strict lockdown in France)	HomeSTEAD	Child eating behaviors, Parental feeding practices, Food shopping motivations	Sixty percent of parents reported at least one change in their feeding practices during lockdown compared to the period before the lockdown; When parents changed their practices, they generally became more permissive: less rules, more soothing with food, more child autonomy. They bought pleasurable and sustainable foods more frequently, prepared more home-cooked meals and cooked more with the child	7
Shirlene et al., 2021, USA [30]	Select parents of children aged 5–11 years from social media(*n* = 197) From the 19th of May until the 17th of June 2020	FPSQ	COVID-19-related perceived stress, Mothers’ dysregulated eating behaviors, Child feeding practices, Body mass index	COVID-19-related perceived stress was positively associated with mother’s BMI and emotional eating; Rewarding their child’s eating and behavior with food were both positively associated with the number of COVID-19 related life changes	7

*CFQ* Child Feeding Questionnaire, *FPSQ* feeding practices and structure questionnaire, *CFPQ* Comprehensive Feeding Practices Questionnaire, *EMA* Ecological Momentary Assessment, *HomeSTEAD* Home Self-administered Tool for Environmental Assessment of Activity and Diet

### Measurement tools

The Child Feeding Questionnaire (CFQ), which is appropriate for parents of children aged 2–11 years [32], is increasingly and extensively used in research of feeding practices or feeding styles. The CFQ includes 7 dimensions: perceived responsibility, perceived parent weight, perceived child weight, concern about child weight (measures parents' perception and perception of weight), restriction, pressure to eat, and monitoring (evaluate the specific feeding behaviors and attitudes of parents). Three of the included investigations employed the CFQ, which demonstrated good internal consistency [25, 27, 28].

Musher-Eizenman developed a Comprehensive Feeding Practices Questionnaire (CFPQ) for children aged 2–8 years [33] by combining the CFQ with the Parental Feeding Style Questionnaire (PFQ). The CFPQ contains 49 items and 12 dimensions. The content becomes more comprehensive after incorporation of the evaluation of positive feeding behavior, such as modeling, teaching about nutrition, and the encouragement of balance and variety. The internal consistency and reliability of the subscales were 0.61–0.93 in the included articles [21].

Jansen et al. [34] developed the feeding practices and structure questionnaire (FPSQ) for mothers of 2-year-old children (21–27 months old), which has 9 dimensions and 40 items. Four of the dimensions (Distrust in Appetite, Reward for Behavior, Reward for Eating, and Persuasive Feeding) reflect nonresponsive feeding practices, and the other five dimensions (structured meal setting, structured meal timing, family meal setting, overt restriction, and covert restrictions) reflect the meal environment and restriction structure. The FPSQ has been validated in infants and toddlers (< 2 years) to track feeding practices from infancy to childhood; a parsimonious version of the FPSQ has been validated in children aged 2–5 years and has proven to be a reliable tool for usage [35, 36].

Vaughn et al. developed the Home Self-administered Tool for Environmental Assessment of Activity and Diet (HomeSTEAD), a brief and comprehensive psychometric evaluation tool for food-nurturing practices for children aged 3–12 years [37]. The tool has 86 items that address coercive control practices, autonomy supportive practices, and structural practices, all of which have good internal reliability (α > 0.62) [37].

Ecological momentary assessment (EMA) is a method of recording subjects' behaviors in real-time using smart devices such as cell phones to minimize recall bias and capture fluctuations in behavior over time more precisely [38]. The Real-Time Parent Feeding Practices Measurement Tool, developed in Loth’s study [26], was used to assess food-related parenting practices in EMA, including the CFQ, CFPQ, and other questionnaires.

### Changes in coercive control

In this review, the features of coercive control include increased restriction (Parent-centered restriction of children's food intake), pressure to eat, threats and bribes(rewards), and the use of food to control negative emotions.

A total of six studies referred to elements of parental coercive control practices. Four studies found increased parental usage of restrictive practices and pressure to eat [26, 27], two of which concluded that the negative effects of COVID-19 on parents and distress would increase feeding restrictions and pressure to eat [25, 29]. According to a longitudinal study [28], parental restricted usage increased from before COVID-19 to T1 (May 2020) and returned to pre-pandemic levels at T2 (September 2020). Two studies found that parents used food to reward behaviors with children than pre-COVID-19, and soothed children with food based on their emotions. Preschoolers’ parents also claimed to support snack parenting practices and general feeding practices [21], and higher COVID-19-specific stress was associated with more emotion-based snack feeding.

### Changes in structural practices

The article reports an increase in monitoring practices (parents concerned about their children's diet), food preparation, meal and snack routines, a decrease in rules and limits (parents prescribing when and how much children should eat), and the implementation of unstructured practices.

Five studies explored structural practice elements; two showed an increase in monitoring, while one found that monitoring utilization declined to pre-COVID-19 levels as the pandemic progressed [27, 28]. There was no difference in monitoring utilization in the context of food security status [28]. Three studies showed household food preparation. Among families, 66% of parents said they would cook more than before; 62% of families would consume less take-out fast food [27] and spend more time cooking with their children; and parents with higher levels of education would buy healthier, more comfortable, and more sustainable foods [31]. However, 56% of households with extremely low food security reported a decrease in fresh food. [27]. The ability of COVID-19 to maintain people's fundamental quality of life reduced supply scarcity, and the amount of fresh and unprocessed food in the household began to increase [28]. Four studies showed that rules and limits on unhealthy foods have been reduced, and feeding practices have become more tolerant of meeting the needs of children (e.g., what and how much to eat, etc.) [21, 26]. The frequency of snacking between meals increased in 36% of children [31], while the total amount of food, high-calorie snacks, and desserts/candy in the household varied depending on food security status [27].

### Changes in autonomy support practices

Two studies on parental autonomy support were reported. According to one research [21], parental autonomy support practices, such as actively encouraging children to participate in food preparation and teaching about nutrition at mealtime have increased. Unlike other studies, this study compared three practices (i.e., positive mealtime practices, general feeding practices, and snack parenting practices) in preschool and school-age children separately. In all, 10 practices were found to be different in the two populations when examining a total of 15 routines. The study found, for example, that preschoolers were less likely to prepare food and their parents were more likely to eat with their children. In another study, the use of autonomy support practices was lower than before COVID-19 [26].

## Discussion

This study attempted to summarize the changes in parental feeding practices during COVID-19, analyze existing and potential problems, and provide behavioral and nutritional guidance to parents and children. The results showed that parental coercive control practices (e.g., pressure to eat, restricted diets, and food rewards) increased during the COVID-19 pandemic, and that structure and autonomy support practices had different outcomes depending on the content of the study (e.g., structural practices in which parents monitored children more but were not overly prescriptive about children's snack intake).

COVID-19 has had negative short- or long-term effects on parents, children, and families [39], resulting in increased levels of stress and depression [40]. Negative parental emotions and stress can affect parents' enthusiasm for feeding practices, leading to an increase in coercive control feeding practices [26, 27]. This is consistent with previous studies [17, 41]. Stress can effectively interfere with parents' ability to observe children's behavior and limit children's ability to regulate their energy intake [29]. Coercive control practices, for example, can reduce vegetable intake over time [42]. Moreover, to alleviate children’s boredom and distress due to COVID-19 or parents' lack of energy to restrict food provision [18, 26], stressed parents use food to compensate for the impact on children’s life aspects [43]; this could also explain why parental stress is associated with increased emotional and snack feeding practices. As a result, parents are less likely to have specific rules or limits on their children's snacks and to provide them on an emotional basis [21], resulting in greater intake of high-calorie foods such as potato chips and sugar-sweetened beverages among children in home isolation [44, 45]. Mothers who experienced greater COVID-19 life changes had more rewarding diet-related behaviors and pressure to eat, and mothers with a high body mass index were more likely to use food to control their child's negative emotions [30]. It has been reported that utilizing snacks as a reward may increase external factors associated with children's diets and may also influence children's eating behaviors by increasing exposure to unhealthy snacks, resulting in childhood overeating and obesity [46]. Although parents provide proper guidance to their children during a pandemic (e.g., explaining nutrition, involving children in daily meal preparation, and encouraging positive and healthy eating habits), it may be difficult for parents to maintain a stable environment to ensure children's health and nutritional support under economic and life stress. As a result, parents experiencing stress during the pandemic can be advised on how to cope with stress and sustain supportive feeding.

In addition, the pandemic’s lockdown policy made fresh fruits and vegetables more difficult to obtain, and food insecurity during covid-19 was cited in all three included studies, with the same results as in previous studies [47, 48]. We found that families with food insecurity used coercive control practices more frequently, including highly stressed parents who may force children to eat to avoid wasting [49] or restrict intake to avoid food consumption [50], causing children to overeat when food is plentiful and affecting their dietary regulation [51]. Meanwhile, children's dietary intake is influenced by their home food environment [52], children living in food insecurity status have poorer availability, affordability, and accessibility to nutritious foods [53]. Parents experience various barriers in implementing structured practices [54], leading to the children receiving poor-quality diet [55], which results in a rise in the incidences of diet-related chronic childhood diseases such as obesity [56]. Despite the relaxation of epidemic prevention measures in many regions, food security issues continue to arise, whether as a result of the outbreak or other economic shocks. Food insecurity has been considered now as detrimental to force-feeding [57, 58]. As educators, we may provide timely and supportive feeding instructions to food insecure families to reduce the negative impact on nutrition from COVID-19 or similar stressful events later in life, which is within our power.

When subsequently creating more rational feeding practices to promote the usage of positive health-related behaviors in children and parents, the age of the child should be considered first. Children had a wide age range throughout the research, with clear distinctions between preschoolers and school-age children. School-age children who acquire a sense of diligence have rapid cognitive and ability development, are more likely to develop healthy eating behaviors, are more inclined to help prepare food at mealtime, and have greater autonomy and skills than preschool children [21, 59]. Preschoolers are more dependent on their parents for food. Since parents have no specific rules or restrictions on food, the use of treats to reward and comfort behaviors is more frequent [60]. Consistent with the findings of Yee's research, praise and rewards were dominant for children aged < 6 years, while setting rules or limits was more effective for children aged > 7 years [4]. There are studies showing that the type of feeding practices parents used is associated with child mood, with positive child mood associated with greater use of autonomy-supportive practices [26], child boredom with parental use of soothing food or less restriction [31]. Parents’ behavior may be affected by their children’s emotions. As a result of the elevated negative emotions of children, parents who want their children to be more active but are unable to achieve their goals are likely to be less restrictive to their children's diets and exhibit more tolerant feeding practices. Both of their emotions have a potential impact on feeding practices. Understanding the two-way link between parent and child may be useful in implementing better feeding practices. Future research should strive to explore the long-lasting effects of COVID-19 on parental feeding practices and children, particularly changes in children's eating habits and eating behaviors, as well as the effects on body weight. Children's sedentary behavior [61], screen time [62], and dietary changes [63] are all likely to increase obesity rates in children in the post-pandemic era. Consequently, it might be beneficial to detect these changes in time for child development.

Although we summarized variations in parental feeding practices in this systematic review, some points need to be considered. First, the included studies did not specifically explore whether feeding practices were associated with child outcomes (e.g., changes in diet, eating behaviors, or body composition) to further explore the impact of changes in parental feeding practices. Second, in the included studies, the CFQ and FPSQ were employed beyond the age-applicable range of the questionnaire and may not have correctly assessed children outside the applicable range. The age range of the children surveyed also spanned a wide range and did not fully reflect the feeding practices of children in a particular age group. In addition, the use of self-report questionnaires in all study design methods may have resulted in parental recall bias, and changes in the current social pressures and the feeding practice dynamics may have been underrepresented. The heterogeneity of the study instruments used in the study made direct comparisons of our results impossible during our integration procedure. A reliable method that investigates differences in parental feeding practices would be potentially valuable to the study of parental feeding practices. Finally, the included literature in the study does not specify the government epidemic policies in place when the studies were conducted. In terms of chronology, almost all participating areas were under lockdown, making our results comparable. However, most of the articles included in this study were from cross-sectional surveys conducted in the United States; therefore, studies from other country regions may be a useful addition to the future research. Nevertheless, this study summarizes the variations in parental feeding practices during COVID-19 and serves as a foundation for further exploration of the long-term impacts of the COVID-19 pandemic on children (e.g., dietary regulation/behavior, body weight).

## Conclusions

This review suggests that parents changed their feeding practices during the COVID-19 pandemic. The findings of increased coercive control and changes in structure and autonomy support practices may assist researchers in further exploring the impact on children's eating habits and healthy eating behaviors, and providing accurate targets for future interventions. The review also attempted to explore the factors that influence parental feeding practices and revealed that stress (whether caused by unemployment, financial instability, or food insecurity) is one of the more critical factors noted during COVID-19. In the post-epidemic age, we must consider and implement measures to assist parents in coping with these challenges and provide guidance on healthy feeding strategies.

## Supplementary Information

**Additional file 1.** 

## Data Availability

The datasets supporting the conclusions of this article are included within the article and its additional files.
