# Supplementary material for: Variation of parental feeding practices during the COVID-2019 pandemic: a systematic review
Source: BMC Public Health. 2022 Aug 23;22:1600. doi: 10.1186/s12889-022-14027-6 (PMC9395808; doi:10.1186/s12889-022-14027-6)
Supplement: Supplementary file 1 — Additional file 1. [file 12889_2022_14027_MOESM1_ESM.docx]

**Supplementary Table A: search strategy**

| **Database** | **Number of results** | **Search terms** |
| --- | --- | --- |
| PubMed | 400 | ((((((((((((((("Feeding Behavior"[Mesh]) OR (Feeding Behavior*[Title/Abstract])) OR (Eating Behavior*[Title/Abstract])) OR (Feeding-Related Behavior*[Title/Abstract])) OR (Feeding Pattern*[Title/Abstract])) OR (Food Habit*[Title/Abstract])) OR (Eating Habit*[Title/Abstract])) OR (Dietary Habit*[Title/Abstract])) OR (Diet Habit*[Title/Abstract])) OR (feeding practice[Title/Abstract])) OR (Feeding status[Title/Abstract])) OR (feeding style[Title/Abstract])) OR (feed*[Title/Abstract]) ) OR (food parenting practices[Title/Abstract])) AND (((((("Child"[Mesh]) OR (child*[Title/Abstract])) OR (adolescen*[Title/Abstract])) OR (teen*[Title/Abstract])) OR (pediatric*[Title/Abstract])) OR (preschool*[Title/Abstract]))) AND (((((((((((((COVID-19[Title/Abstract]) OR (SARS-CoV[Title/Abstract])) OR (covid-2019[Title/Abstract])) OR (SARS-cov-2[Title/Abstract])) OR (coronaviru*[Title/Abstract])) OR (2019-ncov[Title/Abstract])) OR (Pandemic[Title/Abstract])) OR (coronavirus disease 2019[Title/Abstract])) OR (severe acute respiratory syndrome coronavirus 2[Title/Abstract])) OR (2019 novel coronavirus’[Title/Abstract])) OR (2019-nCoV[Title/Abstract])) OR (corona virus[Title/Abstract])) OR (COVID[Title/Abstract])) |
| EMBASE | 481 | ('covid 19':ab,ti OR 'sars cov':ab,ti OR 'covid 2019':ab,ti OR 'sars cov 2':ab,ti OR coronaviru*:ab,ti OR pandemic:ab,ti OR 'coronavirus disease 2019':ab,ti OR 'severe acute respiratory syndrome coronavirus 2':ab,ti OR '2019 novel coronavirus':ab,ti OR '2019 ncov':ab,ti OR 'corona virus':ab,ti OR covid:ab,ti) AND (child*:ab,ti OR adolescen*:ab,ti OR teen*:ab,ti OR pediatric*:ab,ti OR preschool*) AND ('feeding behavior*':ab,ti OR 'eating behavior*':ab,ti OR 'feeding-related behavior*':ab,ti OR 'feeding pattern*':ab,ti OR 'food habit*':ab,ti OR 'eating habit*':ab,ti OR 'dietary habit*':ab,ti OR 'diet habit*':ab,ti OR 'feeding practice':ab,ti OR 'feeding status':ab,ti OR 'feeding style':ab,ti OR feed*:ab,ti) |
| CINAHL | 302 | (‘COVID-19’ OR ‘SARS-CoV’ OR ‘covid-2019’ OR ‘SARS-cov-2’ OR ‘coronaviru*’ OR ‘2019-nCoV’ OR ‘Pandemic’ OR ‘coronavirus disease 2019’ OR ‘severe acute respiratory syndrome coronavirus 2’ OR ‘2019 novel coronavirus’ OR ‘corona virus’ OR COVID) AND (child* OR adolescen* OR teen* OR Pediatric* OR preschool*) AND (‘Feeding Behavior’ OR ‘Feeding Behavior*’ OR ‘Eating Behavior*’ OR ‘Feeding-Related Behavior*’ OR ‘Feeding Pattern*’ OR ‘Food Habit*‘ OR ‘Eating Habit*’ OR ‘Dietary Habit*’ OR ‘Diet Habit*’ OR ‘feeding practice’ OR ‘Feeding status’ OR ‘feeding style’ OR feed* OR ‘food parenting practices’) |
| Web of Science | 434 | ((((((((((((AB=(COVID-19 )) OR AB=(SARS-CoV)) OR AB=(covid-2019)) OR AB=(SARS-cov-2)) OR AB=(coronaviru*)) OR AB=( 2019-ncov)) OR AB=(Pandemic)) OR AB=(coronavirus disease 2019)) OR AB=(severe acute respiratory syndrome coronavirus 2)) OR AB=(2019 novel coronavirus’)) OR AB=(2019-nCoV)) OR AB=(corona virus)) OR AB=(COVID) AND (((((TS=(Child)) OR AB=(child*)) OR AB=((adolescen*)) OR AB=(teen*)) OR AB=(Pediatric*)) OR AB=(preschool*)) AND (((((((((((TS=(Feeding Behavior)) OR AB=(Feeding Behavior*)) OR AB=(Eating Behavior*)) OR AB=(Feeding-Related Behavior*)) OR AB=(Feeding Pattern*)) OR AB=(Food Habit*)) OR AB=(Eating Habit*)) OR AB=(Dietary Habit*)) OR AB=(Diet Habit*)) OR AB=(feeding practice)) OR AB=(Feeding status)) OR AB=(feeding style)) OR AB=(FEED*) |
| Medline | 771 | (‘COVID-19’ OR ‘SARS-CoV’ OR ‘covid-2019’ OR ‘SARS-cov-2’ OR ‘coronaviru*’ OR ‘2019-nCoV’ OR ‘Pandemic’ OR ‘coronavirus disease 2019’ OR ‘severe acute respiratory syndrome coronavirus 2’ OR ‘2019 novel coronavirus’ OR ‘corona virus’ OR COVID) AND (child* OR adolescen* OR teen* OR Pediatric* OR preschool*) AND (‘Feeding Behavior’ OR ‘Feeding Behavior*’ OR ‘Eating Behavior*’ OR ‘Feeding-Related Behavior*’ OR ‘Feeding Pattern*’ OR ‘Food Habit*‘ OR ‘Eating Habit*’ OR ‘Dietary Habit*’ OR ‘Diet Habit*’ OR ‘feeding practice’ OR ‘Feeding status’ OR ‘feeding style’ OR feed* OR ‘food parenting practices’) |

**Supplementary Table B: quality of bias assessment**

|  | **Selection** | | | | **Compatibility** | **Outcome** | | | |
| --- | --- | --- | --- | --- | --- | --- | --- | --- | --- |
| **Study (authors, year, country)** | **Representativenes of the Sample** | **Sample Size** | **Non-Respondents** | **Acertainment of Exposure** | **Based on Design and Analysis** | **Assessment of Outcome** | **Statistical Test** | **Total** | **Risk of Bias** |
| Adams et al, 2020, USA | 1 | 1 | 1 | 1 | 0 | 1 | 1 | 6 | Medium |
| Caroline et al, 2020, USA | 1 | 1 | 1 | 2 | 2 | 1 | 1 | 9 | High |
| Adams et al, 2021, USA | 1 | 1 | 1 | 1 | 0 | 1 | 1 | 6 | Medium |
| Frankel et al, 2021, USA | 1 | 0 | 0 | 2 | 2 | 1 | 1 | 7 | Medium |
| Jansen et al, 2021, USA | 1 | 1 | 1 | 2 | 2 | 1 | 1 | 9 | High |
| Loth et al, 2021, USA | 1 | 0 | 0 | 1 | 2 | 1 | 1 | 6 | Medium |
| Philippe et al, 2021, France | 1 | 1 | 0 | 1 | 2 | 1 | 1 | 7 | Medium |
| Shirlene et al, 2021, USA | 1 | 1 | 1 | 2 | 0 | 1 | 1 | 7 | Medium |
